# SOCIAL AND PSYCHOLOGICAL FACTORS AND COGNITIVE FUNCTION: FINDINGS FROM INTERNATIONAL SURVEYS

**DOI:** 10.1093/geroni/igz038.741

**Published:** 2019-11-08

**Authors:** Hanzhang Xu, Bei Wu

**Affiliations:** 1 Duke University, Durham, North Carolina, United States; 2 New York University, New York, New York, United States

## Abstract

This symposium examines how social and psychological factors including formal schooling, subjective memory, and neuropsychological symptoms impact cognitive function among older adults in China and the U.S. The first paper used the WHO’s Study on global AGEing and adult health Wave-1 data to examine the relationship between subjective cognitive function, perceived memory decline, and objective cognitive function among older adults in China. The results showed worse subjective cognitive function was associated with poorer working memory and verbal fluency, whereas greater perceived memory decline was associated only with poorer working memory. Furthermore, using data from the Health and Retirement Study, the second paper applied group-based trajectory modeling to assess dual trajectories of subjective memory impairment and objective cognitive decline. Four distinct dual-trajectory typologies were identified, suggesting complex co-occurring changes in subjective memory and objective cognition in older adults. The third paper characterized the trajectories of three neuropsychological symptoms (pain, insomnia, and depression) prior to dementia onset. Using data from the National Health and Aging Trends Study, the study found older adults with dementia exhibit distinct trajectory of depression before dementia onset than those without dementia. Trajectories of pain and insomnia did not differ before dementia onset. The last paper examined the effect of education on cognitive decline among lower educated older adults using data from the Longitudinal Study of Older Adults in Anhui Province, China. Results suggest that older adults with some formal schooling had slower cognitive decline; the gap in cognition between the literate and illiterate widened with age.

